# Successful Removal of Portions of Bone, (Probably the Pubis,) with a Large Calculous Mass from the Bladder

**Published:** 1846-11

**Authors:** Paul F. Eve

**Affiliations:** Prof. of Surgery in the Medical College of Georgia


*Successful removal of portions of Bone, (probably the pubis,) with a large calculous mass from the bladder.* By Paul F. Eve, M. D., Prof. of Surgery in the Medical College of Georgia.—In January last, I received a letter from an intellgent physician of this State, who mentioned that while on a recent visit to Alabama, he had seen a negro woman laboring under symptoms of stone, and that he had recommended her to be sent to my Infirmary. Upon the arrival of the patient on the 8th of this month (January), the following history of the case was obtained. She is now twenty-four years old, is married and has borne one child: is of good constitution, well made, and has enjoyed uninterrupted health up to the time of her accident. About four years ago, while in a stable loft, she fell, and in descending got astride a projecting pin, which injured her very seriously. She states that her external organs of generation were greatly contused, and that it was nearly a year before she could walk much or with ease. Her master, writes: "There was great contusion of the soft parts, but a fracture I never could detect. After the fall, she was confined to her bed six months, unable to walk, and for twelve months could not labor." Although her owner is a physician, I do not understand he is a practitioner of medicine; and all will acknowledge how obscure is the diagnosis of fractures of the pelvis. Without, therefore, any reflection whatever upon his intelligence or knowledge of anatomy and surgery, the pubis or ischium, or both, may have been broken by the fall which the patient sustained, without being detected. If no fracture occurred, how account for the long confinement and inability of the patient, even to walk for months? After her recovery, she was hired out, but becoming unable to labour, she had returned home. For the past four months the catamenia have failed, and she has experienced great difficulty and irregularity in urinating. On examining per vaginam, the finger encountered a rough body projecting into that canal through a vesico-vaginal fistula, an inch and a half behind the orifice of the urethra. A sound in the bladder came in contact with a calculus. The urine was bloody, and discharged every few minutes, and could not be retained. The neck of the uterus was soft, irregular and enlarged.

Jan. 9th. A dose of oil was given, which operated well; a warm hip bath was prescribed twice a day, flax-seed tea and moderate diet.

10th. Operated at one o'clock assisted by Drs. Dugas, Means, Martin of Cobham, Shaw of Covington, and in the presence of the Medical Class. As a vesico-vaginal fistula, (so much to be apprehended in lithotomy in the female,) already existed, a groove director was passed through the urethra and fistulous opening into the vagina, and by a probe-pointed bistoury it was cut out. By the section thus made, a finger was introduced into the bladder, and with the calculous forceps, a piece of bone, coated with uric acid, was extracted. This was the foreign body projecting into the vagina, and was irregular in shape, and about three-fourths of an inch square. The bladder was now found filled with a soft calculous deposit, which would allow the blades of the forceps to close through it. After breaking up this mass, another and a larger piece of bone was felt lying behind the pubis. From its position and size, much difficulty was encountered in removing it. The urethra was incised towards the clitoris, and Dr. Dugas also attempted to dislodge it. By the finger it was fortunately thrust from behind the pubis, and then withdrawn in the forceps. This fragment was much more voluminous than the first one extracted, and resembled somewhat the body of the pubis. It was closely impacted in the position described, but the coats of the bladder felt to the finger entire. The only opening in its membranes besides the incisions, being the fistula mentioned, and which was about the size of a rifle ball. Injections were now employed to wash out the bladder, and the patient placed in bed.

The duration of this irregular, and as it is believed singular operation, was nearly an hour, and few thought the patient could so soon or so completely recover. Incontinence of urine from the free incisions in the urethra, as well as from the previously existing vesico-vaginal fistula, was certainly expected.

11th. Called in haste to the patient, at 6, A. M. Found her complaining of pain and soreness throughout the region of the bladder. Prescribed, a tepid bath, morphine, diluent drinks, and repose. In the afternoon she is easier.

12th. Doing remarkably well, even retaining the urine better than could have been expected so soon. Sat up in bed a few minutes.

13th. Is decidedly improving.

17th. Has continued to get better. Examined the bladder, and detected a calculus. Failed to remove it. Failed again on the 18th, when she was instructed to retain her urine as long as possible, and then to pass it while in the hip-bath. A calculous weighing 45 grains was thus expelled on the 20th.

30th. The tip of the finger can only be introduced into the bladder. No foreign body can be detected in it. The urine has become clear and healthy, and the patient can retain it an hour or two. She takes moderate exercise in the house.

February 5th. Has gained much flesh. Complains only of incontinence while walking. She left to-day for the country, preparatory to returning home to Alabama.

The foreign bodies extracted from the bladder in this case, were subjected by Dr. Means, Prof. of Chemistry and Pharmacy, &c., too an analysis. The calculous mass was chiefly composed of uric acid. The pieces supposed to be bone were sawed in two, and presented a cancellated appearance, and were reduced by chemical tests to animal matter and phosphate of lime. I regret not preserving the total weight of all the matter abstracted by the operation.—*Ibid.*

